# Sociodemographic Determinants of Knowledge and Risk Perception Regarding Community Fluoridation: A Cross-Sectional Study in Iași, Romania

**DOI:** 10.3390/epidemiologia7030068

**Published:** 2026-05-12

**Authors:** Catalina Iulia Saveanu, Hociung Roxana, Bogdan Ioan Condrea, Daniela Anistoroaei, Alexandra Ecaterina Saveanu, Maria Sophia Saveanu, Loredana Golovcencu

**Affiliations:** 1 Department of Dent-Alveolar and Maxillo-Facial Surgery, Faculty of Dental Medicine, University of Medicine and Pharmacy Grigore T Popa, 700115 Iasi, Romania; catalina.saveanu@umfiasi.ro (C.I.S.); loredana.golovcencu@umfiasi.ro (L.G.); 2 Faculty of Dental Medicine, University of Medicine and Pharmacy Grigore T Popa, 700115 Iasi, Romania; roxanahociung@yahoo.com (H.R.); saveanu.alexandra-ecaterina@d.umfiasi.ro (A.E.S.); md-rom-10944@students.umfiasi.ro (M.S.S.)

**Keywords:** fluoridation, risk perception, epidemiology, public health, cross-sectional study, oral health literacy

## Abstract

**Background and Objectives:** Community fluoridation is an effective public health measure for dental caries prevention; however, knowledge and risk perception vary. This study assessed knowledge, attitudes, and sociodemographic determinants related to community fluoridation. **Materials and Methods:** A cross-sectional study was conducted in May–June 2023 among 200 adults from Iași, Romania, using a self-administered questionnaire. Chi-square tests were applied. **Results:** Most respondents were familiar with fluoride (94%) and its protective role (91%), but fewer knew fluoridation methods (34%) or dental fluorosis (53%). Educational level was associated with awareness of water fluoridation (χ^2^ = 32.219, *p* < 0.001), and gender with safety perceptions (χ^2^ = 6.031, *p* = 0.049). Perceived toxicity was strongly associated with fluoridation safety attitudes (χ^2^ = 29.116, *p* < 0.001). **Conclusions:** Although general awareness is high, understanding remains limited. Sociodemographic factors influence knowledge and risk perception, highlighting the need for targeted communication.

## 1. Introduction

Community fluoridation remains one of the cornerstone strategies in preventive dentistry and public oral health due to its well-documented effectiveness in reducing dental caries at the population level [1,2]. Multiple community fluoridation strategies are currently implemented worldwide as population-based preventive measures, including fluoridated drinking water, salt, and milk. These approaches differ in terms of feasibility, infrastructure requirements, and target populations. Community water fluoridation is widely recognized as the most effective and sustainable strategy, while salt fluoridation has been successfully implemented in several national programs, and milk fluoridation is frequently used in school-based preventive initiatives. The selection of an appropriate fluoridation strategy depends on local public health policies, socioeconomic context, and existing infrastructure. [2,3]. Among these approaches, community water fluoridation is widely recognized as the most efficient and sustainable method, providing continuous low-dose exposure without requiring individual behavioral compliance [1,2]. The caries-preventive effect of fluoride is primarily mediated through its topical action on dental enamel, influencing the balance between demineralization and remineralization processes [3,4]. Salt fluoridation has demonstrated effectiveness in several Latin American and European public health programs [3], while milk fluoridation is commonly applied in school-based preventive schemes [5]. The World Health Organization recommends adapting fluoridation strategies to local contexts, as all systemic modalities have shown clinically relevant reductions in caries prevalence [2]. Systemic fluoride intake derives mainly from fluoridated drinking water, infant formula, salt, and dietary supplements, while additional contributions arise from dietary sources such as tea, wine, and seafood [3,5]. Following gastrointestinal absorption, fluoride is distributed via the bloodstream and incorporated into mineralized tissues. The formation of fluorapatite, which exhibits increased resistance to acid dissolution, together with the presence of fluoride in saliva, contributes to sustained remineralization at the tooth surface [5].

In recent years, scientific debate has intensified regarding potential systemic effects of fluoride exposure, particularly during sensitive developmental periods [6,7]. Several studies and systematic reviews have reported possible associations between prenatal or early-life fluoride exposure and neurodevelopmental outcomes. A meta-analysis identified a small but statistically significant reduction in IQ associated with fluoride exposure [6], while the 2024 National Toxicology Program monograph emphasized the need for cautious interpretation of existing evidence and further investigation into fluoride-related neurotoxicity [7]. Till et al. highlighted the importance of careful dose management during pregnancy and infancy [8], and Gopu et al. reported suggestive life-course associations between fluoride exposure and cognitive outcomes [9]. Although some studies have raised concerns regarding potential systemic effects of fluoride exposure, the current body of evidence remains insufficient to contraindicate community fluoridation when applied within recommended limits. These findings underscore the importance of monitoring cumulative fluoride intake and addressing public concerns through transparent and evidence-based communication.

From an epidemiological perspective, such scientific debate may influence public knowledge, attitudes, and risk perception related to community fluoridation [3,5,9]. Understanding how populations perceive both the benefits and potential risks of fluoridation is essential for public health surveillance and effective communication strategies. Assessing fluoride-related knowledge and risk perception across sociodemographic groups can provide valuable insight into informational gaps and population-level determinants that may affect acceptance of fluoridation policies.

Chronic excessive fluoride exposure may lead to skeletal fluorosis, particularly in regions with naturally elevated fluoride concentrations in drinking water [10]. Advances in imaging techniques, such as F-18 Fluoride PET/CT, have contributed to a more detailed understanding of fluoride distribution and its systemic metabolic behavior [10]. Although such conditions are geographically limited, they remain relevant in discussions of cumulative fluoride exposure and public risk perception.

Dental fluorosis represents the most frequently observed adverse effect associated with excessive systemic fluoride intake during enamel development in childhood, when teeth are forming. Clinically, it manifests as enamel opacities or, in more severe cases, structural alterations. Preventive approaches include maintaining fluoride concentrations within recommended limits (0.5–1.0 mg/L, adjusted for climatic conditions) and monitoring cumulative exposure from multiple sources, including drinking water, dietary intake, supplements, and dental products [2,5,11]. From a public health perspective, these recommendations aim to balance caries prevention with the minimization of adverse effects. Community water fluoridation remains one of the most extensively studied population-level interventions and is endorsed as safe, cost-effective, and equitable by major health authorities, including the World Health Organization and the Centers for Disease Control and Prevention [2]. Populations exposed to optimally fluoridated water supplies consistently exhibit lower caries prevalence across age groups, independent of socioeconomic status or oral hygiene practices [2].

Alternative community fluoridation strategies, such as salt or milk fluoridation, continue to play an important role in settings where water fluoridation is not feasible due to infrastructural or regulatory constraints [2]. Regardless of the modality employed, community fluoridation is accompanied by ethical considerations related to autonomy, informed consent, and public trust, emphasizing the importance of transparent communication and community engagement.

Reliable, evidence-based information on community fluoridation is available through major scientific databases, including PubMed, Scopus, and Web of Science, as well as through reports issued by international and national health organizations such as the WHO and CDC [1,2]. However, in the contemporary information landscape, social media and digital platforms represent important sources of health-related information, where content quality may vary substantially. The spread of incomplete or misleading information in these environments may contribute to heterogeneous knowledge and divergent risk perceptions regarding fluoridation at the population level [3,5,9]. Nevertheless, variability in public understanding and interpretation of this information may further contribute to divergent perceptions of fluoridation benefits and risks. Previous population-based research conducted by the same research group has addressed public awareness and preventive health behaviors in different medical contexts, including dermato-oncology screening [12].

Study Aim

The aim of this study was to assess the level of knowledge and risk perception regarding community fluoridation among adults residing in Iași Municipality, Romania, and to examine associations between fluoride-related perceptions and selected sociodemographic variables.

Research Hypothesis

There is a significant association between knowledge or risk perception related to community fluoridation and at least one sociodemographic variable (age, gender, educational level, or residential background).

## 2. Materials and Methods

### 2.1. Study Design and Setting

This study employed a questionnaire-based cross-sectional epidemiological design and was conducted between May and June 2023 in Iași, a major urban academic center in northeastern Romania. Iasi is characterized by substantial demographic and educational diversity, making it an appropriate setting for assessing population-level knowledge and risk perception related to community fluoridation.

A semi-structured, multiple-choice questionnaire was developed to assess fluoride-related knowledge and perceptions. To ensure clarity and internal consistency, the instrument was pilot tested on 10 volunteers, and minor refinements were made to item wording, clarity, and overall length based on participant feedback.

According to national census data, the adult population of Iași is approximately 760,774 residents [13]. Sample size estimation was performed using the Cochran formula [14], assuming a 95% confidence level and a ±7% margin of error, which resulted in a minimum required sample of 196 participants. A final sample of 200 respondents was included in the analysis. Internal reliability testing demonstrated acceptable internal consistency (Cronbach’s alpha = 0.759).

### 2.2. Study Sample

The target population consisted of adults aged 18 years and older residing in Iași. A total of 200 adults voluntarily participated in the study. Participants were recruited using a non-probability convenience sampling approach, through online dissemination of the questionnaire via social media and academic networks within Iași. Participation was voluntary.

Inclusion criteria included adults aged ≥18 years residing in Iași, with the ability to understand and complete the questionnaire. Exclusion criteria included incomplete responses and failure to provide informed consent.

All participants were assured of complete anonymity, and no academic, professional, or institutional consequences were associated with participation. Participants were informed that the study aimed to improve understanding of fluoride-related knowledge and perceptions at the population level.

### 2.3. Study Instrument Development

Data were collected using an 18-item structured questionnaire designed to balance comprehensiveness with accessibility. The questionnaire was administered electronically using a secure Google Forms platform. Access was provided via unique links, and anonymized identification codes were assigned to each participant to ensure confidentiality.

The study adhered to established ethical principles, including voluntary participation, anonymity, and responsible data handling. Participants received a brief explanation of the study objectives and procedures prior to participation. Eligible respondents included adults residing in Iași.

The questionnaire was developed based on a review of the literature and expert input. A pilot test was conducted on 10 participants to ensure clarity and content validity. Internal consistency was assessed using Cronbach’s alpha (0.759), indicating acceptable reliability.

### 2.4. Questionnaire Contents

The questionnaire comprised two main sections designed to capture sociodemographic characteristics and fluoride-related knowledge and perceptions.

Section 1: Sociodemographic Characteristics

The following variables were collected:

Q1: Age

Q2: Gender

Q3: Highest educational level

Q4: Residential background

These variables were used to stratify analysis examining differences in knowledge and risk perception related to community fluoridation.

Section 2: Knowledge and Perceptions Regarding Community fluoridation

This section includes 12 multiple-choice items assessing knowledge and perceptions related to general and community fluoridation, along with one item addressing dental service utilization. The items covered the following domains:

Q5: Knowledge of the protective role of fluoride;

Q6: Awareness of different fluoridation methods;

Q7: Familiarity with the concept of water fluoridation;

Q8: Awareness of the benefits of fluoridated water;

Q9: Perceived need for fluoridated drinking water;

Q10: Familiarity with the term “fluoride”;

Q11: Knowledge of dental enamel fluorosis;

Q12: Perceived personal benefits of fluoride;

Q13: Awareness of benefits and potential risks associated with fluoride supplementation in children;

Q14: Knowledge of reliable information sources regarding fluoride;

Q15: Timing of the most recent dental visit;

Q16: Perceived safety of general fluoridation;

Q17: Perception of fluoride in drinking water as a potential toxic agent.

Collectively, these items were designed to assess both conceptual knowledge and population-level risk perception related to community fluoridation.

### 2.5. Statistical Analysis

Data were collected, processed, and analyzed using IBM SPSS Statistics version 26 (IBM Corp., Armonk, NY, USA). Descriptive analysis included frequencies, means, standard deviations, and cross-tabulations. Confidence intervals were calculated for key variables where appropriate.

Inferential analysis included Pearson’s chi-square test to assess associations between categorical variables. Effect sizes were estimated using Phi coefficients, Cramer’s V, and contingency coefficients, as appropriate. Spearman’s rank correlation and Pearson’s correlation coefficient were applied to evaluate associations involving numerical variables. A two-sided *p*-value ≤ 0.05 was considered statistically significant. For variables with ordinal response categories, additional correlation analyses (Spearman’s rank correlation) were considered to better reflect the direction and strength of associations.

### 2.6. Ethical Considerations

The study was conducted in accordance with institutional and international ethical standards for research involving human participants. Written informed consent was obtained from all participants prior to inclusion. Consent documentation outlined the study objectives, procedures, potential risks, and anticipated benefits.

Participant confidentiality and anonymity were strictly maintained. All personal identifiers were removed and replaced with unique numeric codes, and data were stored on encrypted servers accessible only to the research team. Participants received an information sheet describing the purpose of the study and the relevance of their responses for understanding population-level knowledge and perceptions related to community fluoridation.

The research protocol received ethical approval under reference number 307/16.05.2023. Informed consent was obtained electronically prior to participation through the online questionnaire platform. Participants were required to confirm their consent before accessing the questionnaire.

## 3. Results

### 3.1. Demographic Characteristics of Respondents

As shown in Table 1, the study sample was predominantly composed of young adults, with 91% of participants aged 20–30 years. Most respondents were female (82.5%), while males accounted for 16.5%.

Most participants resided in urban areas (61.5%), and the overall level of education was high, with the majority having completed high school (67.5%) or holding a bachelor’s degree (27.5%).

### 3.2. Assessment of Knowledge and Risk Perception Regarding Community Fluoridation

As shown in Table 2, most respondents were familiar with fluoride (94%) and recognized its protective role in oral health (91%). However, knowledge of community fluoridation methods was more limited (34%), and only 53% were aware of dental fluorosis.

Educational level was significantly associated with familiarity with water fluoridation (χ^2^ = 32.219, *p* < 0.001), while gender was associated with awareness of its benefits (χ^2^ = 6.031, *p* = 0.049).

Additional indicators of knowledge were lower, with 40% of respondents reporting awareness of appropriate fluoride use and 49% identifying reliable information sources.

No significant associations were observed between age or place of residence and fluoride-related knowledge.

### 3.3. Demographic Differences in Attitudes Toward Water Fluoridation Safety and Perceived Toxicity

As shown in Table 3, most participants reported a recent dental visit, with no significant associations between dental attendance and sociodemographic variables.

The majority of respondents fully or partially agreed that fluoridation is safe, and no significant differences were observed across sociodemographic groups.

In contrast, perceptions of fluoride toxicity showed significant variation. Age (χ^2^ = 23.736, *p* = 0.022) and gender (χ^2^ = 20.275, *p* = 0.009) were significantly associated with perceived toxicity.

Given the ordinal nature of the response categories, the results should be interpreted with caution, and correlation analyses were considered to better reflect the direction of associations.

### 3.4. Perceptions of Fluoride Toxicity and Public Awareness of Community Fluoridation in a Pediatric Health Context

As shown in Table 4, most respondents were familiar with the term “fluoride” and recognized its protective role in oral health, with no significant association between these indicators and perceived fluoride toxicity (χ^2^ = 2.212, *p* = 0.645; χ^2^ = 4.989, *p* = 0.287).

In contrast, a significant association was observed between perceived fluoride toxicity and attitudes toward the safety of fluoridation (χ^2^ = 29.116, *p* < 0.001).

### 3.5. Sociodemographic Differences in Dental Check-Up Frequency

As shown in Table 5, 32% of respondents reported a dental visit within the previous six months. No significant association was observed between place of residence and dental attendance (χ^2^ = 4.295, *p* = 0.415).

Similarly, educational level was not significantly associated with dental check-up frequency (χ^2^ = 8.568, *p* = 0.104).

### 3.6. Correlations Between Public Knowledge of Fluoride Terminology and Perceived Benefits of Water Fluoridation in the Context of Child Oral Health

As shown in Table 6, most respondents who were unfamiliar with fluoridation types were nevertheless familiar with the term “fluoride” (90.9%), with a significant association between these variables (χ^2^ = 10.305, *p* = 0.016). Awareness of the need for water fluoridation was strongly associated with recognition of its benefits (χ^2^ = 113.46, *p* < 0.001).

### 3.7. Association Between Knowledge of Dental Fluorosis and Attitudes Toward Systemic Fluoride Use

As shown in Table 7, a significant association was identified between familiarity with dental enamel fluorosis and knowledge of appropriate fluoride use (χ^2^ = 67.31, *p* < 0.001).

A significant association was also observed between awareness of reliable information sources and knowledge of appropriate fluoride use (χ^2^ = 55.61, *p* < 0.001).

In contrast, no significant association was found between familiarity with dental fluorosis and perceptions of fluoridation safety (χ^2^ = 8.72, *p* = 0.117).

## 4. Discussions

### 4.1. Scientific Consensus and Controversies Surrounding Community Fluoridation

Community water fluoridation remains a central topic in public health policy and epidemiological research due to its established role in population-level caries prevention. The consistent endorsement by major international health organizations reflects a broad scientific consensus regarding its safety, effectiveness, and contribution to reducing oral health inequalities across populations [1,15,16].

At the same time, the persistence of scientific debate concerning potential adverse effects of fluoride exposure highlights an important tension between established public health benefits and emerging concerns related to cumulative exposure. While current evidence does not contraindicate fluoridation within recommended thresholds, ongoing research into dose–response relationships and potential neurodevelopmental outcomes continue to shape both scientific discourse and public perception [17,18].

From an epidemiological perspective, this coexistence of institutional consensus and public controversy represents a key determinant of population-level risk perception. The amplification of concerns through advocacy groups and public discourse may contribute to heterogeneous attitudes toward fluoridation, even in the presence of strong scientific support [19].

These dynamics underscore the importance of effective, transparent, and evidence-based risk communication, which plays a critical role in maintaining public trust and supporting informed decision-making regarding fluoridation policies.

### 4.2. Assessment of Knowledge and Perceptions Regarding Community Fluoridation

These findings should be interpreted in the broader context of multiple community fluoridation strategies (water, salt, and milk), which may influence public awareness differently depending on their level of implementation and visibility within a given population.

The findings of this study indicate that, although general awareness of fluoride and its protective role in oral health is high, this knowledge remains largely superficial and does not extend to a comprehensive understanding of community fluoridation practices. This discrepancy between general awareness and applied knowledge suggests the presence of gaps in functional oral health literacy, a pattern also reported in previous population-based studies [20,21,22].

The limited understanding of fluoridation modalities observed in this study, despite widespread familiarity with fluoride, indicates that public knowledge may be fragmented and insufficiently integrated into a coherent conceptual framework. Similar patterns have been described in the literature, suggesting that awareness of fluoride benefits does not necessarily translate into understanding of its delivery mechanisms or public health applications [3,9].

The strong association between educational levels and familiarity with water fluoridation highlights the role of formal education as a key determinant of health literacy. This finding reinforces existing evidence that educational attainment influences both access to and interpretation of health-related information [23]. In contrast, the observed gender differences in awareness of fluoridation benefits may reflect variations in health information-seeking behavior or exposure to preventive health messages, as suggested in previous studies [20].

Furthermore, the relatively low level of awareness regarding dental fluorosis and appropriate fluoride use indicates that knowledge of potential risks and safe exposure thresholds remains limited. This imbalance between perceived benefits and understanding of potential adverse effects may contribute to inconsistent or ambivalent attitudes toward fluoridation, as previously reported [5].

Overall, these findings suggest that public understanding of fluoridation is characterized by a distinction between basic recognition and deeper, functional knowledge. From an epidemiological perspective, such disparities in knowledge may influence risk perception and acceptance of fluoridation policies at the population level.

Although the questionnaire used in this study was specifically developed to reflect the local context and demonstrated acceptable internal consistency (Cronbach’s alpha = 0.759), the use of a non-standardized instrument may limit comparability with other studies. This should be considered when interpreting the findings and situating them within the broader literature.

### 4.3. Demographic Variations in Attitudes Toward Water Fluoridation Safety and Toxicity

The findings suggest that engagement with preventive dental services is relatively consistent across sociodemographic groups within the study population, indicating that access to or utilization of dental care may not be strongly differentiated by age, gender, educational level, or place of residence in this context. This relative uniformity may reflect a shared baseline orientation toward preventive oral health behaviors in an urban population.

In contrast, attitudes toward the safety of community fluoridation appear to be broadly positive and relatively homogeneous across demographic categories. This pattern is consistent with previous studies reporting higher levels of acceptance of fluoridation in populations with greater exposure to oral health information and preventive care [24].

However, perceptions of fluoride toxicity demonstrate a different pattern, with significant variation across demographic groups. The observed associations with age and gender indicate that risk perception is not uniformly distributed within the population but is instead shaped by specific sociodemographic characteristics. Higher levels of concern among younger adults and female respondents may reflect increased sensitivity to environmental and health-related risks, as reported in previous research [9,25].

From an epidemiological perspective, this divergence between general acceptance of fluoridation and concern regarding potential toxicity suggests the presence of underlying cognitive and perceptual inconsistencies. Such patterns may contribute to ambivalent attitudes toward fluoridation policies, particularly in contexts where information is incomplete or perceived as conflicting.

Overall, these findings highlight the importance of considering sociodemographic variability in risk perception when designing public health communication strategies. Tailored approaches that address specific concerns within population subgroups may be necessary to ensure consistent understanding and acceptance of fluoridation as a preventive measure.

### 4.4. Perceptions of Fluoride Toxicity and Public Awareness of Community Fluoridation

The findings highlight a complex relationship between fluoride-related knowledge and risk perception. Although general awareness of fluoride and its protective role in oral health is high, this knowledge appears to be largely independent of perceptions regarding fluoride toxicity. This suggests that basic fluoride literacy alone does not necessarily influence how individuals evaluate potential risks.

In contrast, the strong association between perceived toxicity and beliefs regarding the safety of community water fluoridation indicates that attitudes toward fluoridation are more closely linked to risk perception than to factual knowledge. The coexistence of positive beliefs about fluoride benefits with uncertainty or skepticism regarding its safety reflects a pattern of cognitive dissonance in public attitudes.

Similar discrepancies between knowledge and acceptance of fluoridation have been reported in previous studies, suggesting that awareness of benefits does not automatically translate into support for fluoridation policies [3,9]. This pattern underscores the complexity of public understanding, where perceptions are shaped not only by knowledge but also by beliefs, concerns, and broader informational contexts.

From an epidemiological perspective, these findings indicate that the dissemination of information alone may be insufficient to address public concerns. Effective public health strategies should therefore integrate both knowledge-based interventions and approaches that directly address risk perception, including transparent communication of benefits, risks, and existing scientific uncertainties.

Furthermore, the sustainability of community fluoridation programs is closely linked to public trust, institutional credibility, and the broader sociopolitical context. In many settings, public perception and ethical considerations have played a more significant role in shaping fluoridation policies than scientific disagreement alone. Understanding these dynamics is essential for developing evidence-informed and socially responsive public health strategies.

### 4.5. Sociodemographic Determinants of Dental Check-Up Frequency

The findings suggest that recent dental attendance is not strongly differentiated across sociodemographic groups within this study population. The absence of significant associations with residential background and educational level indicates that access to or utilization of dental services may be relatively uniform in this urban context.

This pattern suggests that structural determinants such as place of residence and education may play a less prominent role in shaping dental attendance behavior than is often assumed. Instead, individual-level factors—such as perceived need for care, health awareness, and personal attitudes toward preventive practices—may be more influential in determining dental service utilization.

These results are consistent with previous research indicating that the relationship between sociodemographic characteristics and dental attendance is complex and context dependent. While urban residency and higher educational attainment are frequently associated with improved access to care, their effect on actual utilization may be attenuated by behavioral and psychosocial factors [20,25]. Conversely, some studies have reported stronger associations between education and preventive dental behaviors, highlighting variability across populations and settings [26].

From an epidemiological perspective, these findings emphasize the importance of considering both structural and individual determinants when analyzing patterns of healthcare utilization. Understanding this interplay is essential for designing effective interventions aimed at improving preventive dental attendance at the population level.

### 4.6. Correlations Between Knowledge of Fluoride Terminology and Perceived Benefits of Water Fluoridation

The findings highlight a clear distinction between recognition of fluoride-related terminology and a deeper understanding of fluoridation concepts. The observed discrepancy between familiarity with the term “fluoride” and knowledge of fluoridation modalities suggests that public understanding is often superficial and not fully integrated into a coherent conceptual framework.

In contrast, the strong association between awareness of the need for water fluoridation and recognition of its benefits indicates that conceptual understanding plays a key role in shaping positive attitudes toward fluoridation. This suggests that individuals who grasp the rationale behind fluoridation are more likely to perceive its public health value.

These results are consistent with previous studies reporting fragmented and uneven fluoride-related knowledge, where general awareness does not necessarily translate into accurate or comprehensive understanding [3,5,9]. Variability in knowledge has been linked to differences in educational background, access to information, and exposure to health communication sources.

From an epidemiological perspective, these findings emphasize that effective public health communication should move beyond simple dissemination of terminology and focus on improving conceptual understanding. Strengthening functional fluoride literacy may contribute to more consistent risk perception and greater acceptance of community fluoridation as a preventive strategy.

### 4.7. Correlations Between Knowledge of Dental Fluorosis and Attitudes Toward Systemic Fluoride Use

The findings indicate that more specific fluoride-related knowledge, particularly awareness of dental fluorosis, is associated with a higher level of functional understanding regarding appropriate fluoride use. This suggests that knowledge of potential adverse effects may enhance individuals’ ability to interpret and apply preventive recommendations more accurately.

At the same time, the observed association between access to reliable information sources and improved understanding of fluoride benefits highlights the critical role of credible information in shaping public knowledge. These results reinforce previous evidence emphasizing that both content and source of information are key determinants of health literacy [5,11].

However, the lack of association between fluorosis awareness and perceived safety of community fluoridation indicates that technical knowledge alone does not necessarily translate into positive attitudes toward public health interventions. This dissociation reflects a pattern of cognitive ambivalence, in which individuals may simultaneously recognize both benefits and potential risks without forming a consistent evaluative stance. Similar patterns have been reported in previous studies [3,9,27,28,29,30,31].

From an epidemiological perspective, these findings suggest that public understanding of fluoridation is not only incomplete but also internally inconsistent. Fragmented knowledge—characterized by recognition of terminology without deep conceptual integration—may contribute to uncertainty and variability in attitudes toward fluoridation. This is consistent with previous research documenting gaps between basic awareness and applied understanding of fluoride safety and delivery mechanisms [5,23].

These results further highlight the role of education as a key determinant of fluoride-related knowledge, while also underscoring that knowledge alone may be insufficient to ensure consistent acceptance of fluoridation policies. Broader factors, including trust in public institutions, exposure to conflicting information, and sociopolitical context, are likely to influence public attitudes.

Ultimately, the sustainability and acceptance of community fluoridation programs depend not only on scientific evidence but also on public perception, trust, and effective communication. Understanding these dynamics is essential for the development of evidence-informed and socially responsive public health strategies [1].

### 4.8. Study Limitations

Several limitations should be considered when interpreting the findings of this study. First, the study population was predominantly composed of highly educated individuals residing in an urban setting. This demographic profile may have contributed to the relatively high level of general fluoride awareness observed and limits the generalizability of the results to the broader Romanian adult population, particularly to groups with lower educational attainment or reduced access to health-related information.

Second, although the final sample size (N = 200) slightly exceeded the minimum calculated requirement (N = 196), the narrow margin may have limited statistical power for certain subgroup analyses. In addition, the age distribution was markedly skewed towards young adults aged 20–30 years (91%), which does not reflect the demographic structure of the general adult population and may restrict external validity.

Methodological limitations should also be acknowledged. Data were collected using a self-administered questionnaire, which is inherently susceptible to response bias, including socially desirable responding and subjective interpretation of survey items. Furthermore, the cross-sectional design allows the identification of associations but does not allow for causal inference.

Geographic limitations represent another constraint. Data collection was conducted in a single urban center, limiting representativeness across regions. Future research employing multicenter designs that include both urban and rural populations would enhance external validity.

The use of a non-probability convenience sampling approach may limit the representativeness of the sample and restrict the generalizability of the findings to the broader population. Additionally, the questionnaire-based design may be subject to response bias, including socially desirable responses and variability in participants’ interpretation of survey items.

Finally, this study should be regarded as hypothesis-generating. The findings provide a basis for larger, nationally representative investigations aimed at examining fluoride-related knowledge, risk perception, and attitudes across various population subgroups. Longitudinal designs and stratified sampling approaches would further improve understanding of how fluoride literacy and risk perception evolve over time and influence public acceptance of fluoridation policies.

Overall, these findings highlight the importance of improving fluoride-related knowledge and addressing inconsistencies in risk perception at the population level. Previous research has also explored knowledge, attitudes, and practices related to fluoride use among young adults, emphasizing the role of preventive strategies and health education in this population [27,28,29,30,31].

## 5. Conclusions

This study demonstrates a generally high level of public awareness regarding the benefits of fluoride for dental health, while also identifying substantial gaps in more advanced understanding of community fluoridation, including delivery methods, intake considerations, and safety-related aspects. Although most participants recognized fluoride’s protective role against dental caries, knowledge concerning fluoridation modalities and potential adverse effects, such as dental fluorosis, remained limited.

The statistically significant associations observed between educational level and familiarity with concepts such as water fluoridation, as well as gender-related differences in perceptions of safety, indicate that sociodemographic factors play an important role in shaping fluoride-related knowledge and risk perception. The coexistence of acknowledged dental benefits and concern regarding potential toxicity reflects cognitive ambivalence, highlighting the complexity of public attitudes toward community fluoridation.

These findings underscore the importance of evidence-based public health communication strategies that account for sociodemographic variation in knowledge and perceptions. Effective communication should address not only informational gaps but also underlying concerns, while reinforcing the scientific consensus articulated by authoritative institutions such as the World Health Organization and the Centers for Disease Control and Prevention.

As an exploratory study conducted in a localized urban setting, the results provide a foundation for future population-based research. Larger, nationally representative studies employing stratified sampling designs are warranted to further assess public understanding, risk perception, and acceptance of community fluoridation across diverse social, educational, and geographic contexts. Such investigations would support the development of more informed and context-sensitive public health strategies related to fluoridation policy.

## Figures and Tables

**Table 1:** Demographic characteristics of the study participants (Q1–Q4).

Demographic Data		% (N)
Q1	20–30	91 (182)
	30–40	4 (8)
	40–50	3 (6)
	50–60	2 (4)
Q2	F	82.5 (165)
	M	16.5 (33)
	O	1 (2)
Q3	M	0.5 (1)
	H	67.5 (135)
	Bd	27.5 (55)
	Md	4.5 (9)
Q4	U	61.5 (123)
	R	38.5 (77)

N = count; Q1 = Age, Q2 = Gender, Q3 = Highest level of education completed and Q4 = Background; F = Female; M = Male; O = Others; M = Middle school; H = Highschool; Bd = bachelor’s degree; Md = master’s degree; U = Urban; R = Rural.

**Table 2:** Distribution of responses for assessment of perceived knowledge of general fluoridation.

Question	Answer		Age		Gender		Education level		Background	
		% (N)	*χ^2^*	*p*	*χ^2^*	*p*	*χ^2^*	*p*	*χ^2^*	*p*
Q5	Yes	91 (182)	1.838	0.607 ^a,b^	4.455	0.108 ^a,b^	0.15	0.985 ^a,b^	0.295	0.587
	No	9 (18)								
Q6	Yes	34 (68)	4.576	0.206 ^a^	1.491	0.475 ^a,b^	6.538	0.088 ^a,b^	0.447	0.504
	No	66 (132)								
Q7	Yes	66 (131)	1.186	0.978 ^a,b^	0.198	0.995 ^a,b^	32.219	0.000 ^a,b,^*	1.075	0.584 ^a^
	No	35 (69)								
Q8	Yes	54 (108)	0.648	0.885 ^a^	6.031	0.049 ^a,b,^*	3.109	0.375 ^a,b^	1.783	0.182
	No	46 (92)								
Q9	Yes	53 (106)	1.052	0.789 ^a^	2.294	0.318 ^a,b^	3.484	0.323 ^a,b^	0.669	0.413
	No	47 (94)								
Q10	Yes	94 (188)	0.034	0.998 ^a^	1.167	0.558 ^a,b^	4.299	0.231 ^a,b^	2.861	0.091
	No	4 (7)								
	No, but I would like to know	3 (5)								
Q11	Yes	53 (106)	10.358	0.110 ^a,b^	2.547	0.636 ^a,b^	5.255	0.512 ^a,b^	1.231	0.54
	No	24 (47)								
	No, but I would like to know	24 (47)								
Q12	Yes	40.5 (81)	6.338	0.386 ^a,b^	5.372	0.251 ^a,b^	5.297	0.506 ^a,b^	0.308	0.857
	No	32.5 (65)								
	No, but I would like to know	28 (54)								
Q13	Yes	40 (80)	6.742	0.345 ^a,b^	5.456	0.244 ^a,b^	10.504	0.105 ^a,b^	1.943	0.378
	No	32 (64)								
	No, but I would like to know	28 (56)								
Q14	Yes	49 (98)	5.386	0.495 ^a,b^	7.028	0.134 ^a,b^	6.854	0.335 ^a,b^	2.299	0.317
	No	23 (45)								
	No, but I would like to know	29 (57)								

^a,b^ indicate subgroup comparisons; * *p* < 0.05 statistically significant. Abbreviations: χ^2^—chi-square; *p*—significance level. Q5. Did you know that fluoride protects your teeth? Q6. Do you know how many types of fluoridation there can be? Q7. Are you familiar with the term ‘water fluoridation’? Q8. Are you aware of the benefits of fluoridated water? Q9. Are you aware of the need for drinking water fluoridation? Q10. Are you familiar with the term ‘fluoride’? Q11. Are you familiar with the term ‘dental enamel fluorosis’? Q12. Do you know how you can benefit from fluoride? Q13. Are you aware of the benefits of fluoride in supplements intake and minimizing the risk of fluorosis for your child? Q14. Do you know where you can find more reliable information about fluoride?

**Table 3:** Distribution of responses with multiple answer data about assessment of knowledge of general fluoridation.

Question		% (N)	Age		Gender		Education Level		Background	
			*χ^2^*	*p*	*χ^2^*	*p*	*χ^2^*	*p*	*χ^2^*	*p*
Q15	1 Week	11 (21)	13.21	0.586	7.491	0.678	22.15	0.104	5.005	0.415
	1 Month	23 (45)								
	6 Months	32 (64)								
	1 Year	20 (40)								
	2 Years	6 (12)								
	3 Years	9 (18)								
Q16	Total agreement	22 (43)	12.2	0.429	13.04	0.110 ^a,b^	8.815	0.719	3.753	0.44
	Partial agreement	48 (95)								
	Neutral	23 (46)								
	Total disagreement	7 (14)								
	Partial disagreement	1 (1)								
Q17	Total agreement	2 (4)	23.74	0.022 *	20.28	0.009 *	13.08	0.363	2.594	0.628
	Partial agreement	13 (26)								
	Neutral	35 (70)								
	Total disagreement	36 (72)								
	Partial disagreement	14 (28)								

* χ^2^ = Chi-square test; *p* = significance level, *p* < 0.05 indicates statistically significant association. ^a,b^ indicate statistically significant differences between specific subgroups (post hoc comparisons). *r* = Pearson’s R; *p* = Significance level; Q15 = Time of last dental check-up; Q16 = Given the dental health benefits, do you think general fluoridation is safe? Q17 = Could the fluoride supply to the community through water fluoridation be considered a toxic substance?

**Table 4:** Comparative crosstabulation analysis between responses to Q17 (Could the fluoride supplied to the community through water fluoridation be considered a toxic substance?) and respondents’ responses to Q10 (Are you familiar with the term “fluoride”?), Q5 (Did you know that fluoride protects your teeth?), and Q16 (Given the dental health benefits, do you think general fluoridation is safe?).

		TA	PA	NT	PD	TD	Total	*r*	*p*
		% (N)	% (N)	% (N)	% (N)	% (N)	% (N)		
Q17									
Q10	Yes	2 (4)	12 (24)	32 (63)	35 (69)	14 (28)	94 (188)	−0.088	0.645
	No		0.5 (1)	2.5 (5)	0.5 (1)		3.5 (7)		
	No, but I would like to know		0.5 (1)	1 (2)	1 (2)		2.5 (5)		
	Total	2 (4)	13 (26)	35 (70)	36 (72)	14 (28)	100 (200)		
Q5	Yes	2 (4)	11 (22)	31 (62)	33 (66)	14 (28)	91 (182)	−0.118	0.095
	No		2 (4)	4 (8)	3 (6)		9 (18)		
	Total	2 (4)	13 (26)	35 (70)	36 (72)	14 (28)	100 (200)		
Q16	Total agreement	1.5 (3)	1.5 (3)	5.5 (11)	4.5 (9)	9 (18)	22 (44)	−0.343	0.00
	P = Partial agreement	0.5 (1)	5 (10)	14 (28)	24 (47)	4.5 (9)	47.5 (95)		
	NT = neutral		2 (4)	15 (29)	6 (12)	0.5 (1)	23 (46)		
	TD = Total disagreement		4 (8)	1 (2)	2 (4)		7 (14)		
	PD Partial disagreement	0.5 (1)					0.5 (1)		
	Total	2 (4)	12.5 (25)	35 (70)	36 (72)	14 (28)	100 (200)		

*r* = Pearson’s R;  *p* = Significance level, 1 W = One week ago; 1 M = One month ago; 6 M = Six months ago; 1 Y = One year ago; 2 Y = Two years ago; 3 Y = Three years ago; TA = Total agreement; PA = Partial agreement; NT = Neutral; PD = Partial disagreement; TD = Total disagreement; Q17 = Could the fluoride supply to the community through water fluoridation be considered a toxic substance? Q10 = Are you familiar with the term “fluoride”? Q5 = Did you know that fluoride protects your teeth? Q16 = Given the dental health benefits, do you think general fluoridation is safe?

**Table 5:** Comparative crosstabulation analysis between Q4 (Background), Q3 (Highest degree of education completed), and Q15 (Time of last dental check-up).

		1 W	1 M	6 M	1 Y	2 Y	3 Y	Total	*r*	*p*
		(%) N	(%) N	(%) N	(%) N	(%) N	(%) N	(%) N		
Q15										
Q3	Middle school					0.5 (1)		0.5 (1)	−0.039	0.582
	Highschool	6.5 (13)	16.5 (33)	21 (42)	14 (28)	2.5 (5)	7 (14)	67.5 (135)		
	Bachelor’s degree	3.5 (7)	4.5 (9)	10 (20)	5 (10)	2.5 (5)	2 (4)	27.5 (55)		
	Master’s degree	0.5 (1)	1.5 (3)	1 (2)	1 (2)	0.5 (1)		4.5 (9)		
	Total	10.5 (21)	22.5 (45)	32 (64)	20 (40)	6 (12)	9 (18)	100 (200)		
Q4	Urban	5.5 (11)	12 (24)	23 (46)	12 (24)	3.5 (7)	5.5 (11)	61.5 (123)	−0.045	0.582
	Rural	5 (10)	10.5 (21)	9 (18)	8 (16)	2.5 (5)	3.5 (7)	38.5 (77)		
	Total	10.5 (21)	22.5 (45)	32 (64)	20 (40)	6 (12)	9 (18)	100 (200)		

*r* = Pearson’s R; *p* = Significance level; 1 W = One week ago; 1 M = One month ago; 6 M = Six months ago; 1 Y = One year ago; 2 Y = Two years ago; 3 Y = Three years ago; Q3 = Highest level of education completed; Q4 = Background; Q15 = Time of last dental check-up.

**Table 6:** Comparative crosstabulation analysis between responses to Q6 (Do you know how many types of fluoridation there can be?) and Q7 (Are you familiar with the term “fluoride”?), Q8 (Are you aware of the benefits of fluoridated water?), and Q9 (Are you aware of the need for drinking water fluoridation?).

		Yes	No	Total	*r*	*p*
		(%) N	(%) N	(%) N		
	Q6					
Q7	Yes	34 (68)	60 (120)	94 (188)	0.171	0.016
	No		3.5 (7)	3.5 (7)		
	No, but I would like to know;		2.5 (5)	2.5 (5)		
	Total	34 (68)	66 (132)	100 (200)		
	Q9					
Q8	Yes	47.5 (95)	6.5 (13)	54 (108)	0.759	0.00
	No	5.5 (11)	40.5 (81)	46 (92)		
	Total	53 (106)	47 (94)	100 (200)		

*r* = Pearson’s R; *p* = Significance level; Q6 = Do you know how many types of fluoridation there can be? Q7 = Are you familiar with the term ‘water fluoridation’? Q8 = Are you aware of the benefits of fluoridated water? Q9. Are you aware of the need for drinking water fluoridation?

**Table 7:** Comparative crosstabulation analysis between responses to Q11 (Are you familiar with the term “dental enamel fluorosis”?), Q13 (Do you know how you can benefit from fluoride intake and minimize the risk of fluorosis for your child?), Q14 (Do you know where you can find more reliable information about fluoride?), and Q16 (Given the dental health benefits, do you think general fluoridation is safe?).

		Yes	No	Nk	Total	*r*	*p*
		% (N)	% (N)	% (N)	% (N)		
	Q14						
Q13	Yes	33.5 (67)	5.5 (11)	40 (2)	40 (80)	0.66	0.000 c
	No	10 (20)	16 (32)	32 (12)	32 (64)		
	Nk	5.5 (11)	1 (2)	28 (43)	28 (56)		
	Total	49 (98)	22.5 (45)	100 (57)	100 (200)		
	Q11						
Q16	TA	15 (30)	3 (6)	21.5 (7)	21.5 (43)	0.12	0.101 c
	PA	23 (46)	12.5 (25)	47.5 (24)	47.5 (95)		
	NT	9 (18)	6.5 (13)	23 (15)	23 (46)		
	TD	5 (10)	1.5 (3)	7 (1)	7 (14)		
	PD	0.5 (1)		0.5 (0)	0.5 (1)		
	Total	52.5 (105)	23.5 (47)	99.5 (47)	99.5 (199)		
	Q13						
Q11	Yes	38.5 (77)	8 (16)	53 (13)	53 (106)	0.69	0.000 c
	No	1.5 (3)	18.5 (37)	23.5 (7)	23.5 (47)		
	Nk		5.5 (11)	23.5 (36)	23.5 (47)		
	Total	40 (80)	32 (64)	100 (56)	100 (200)		

*r* = Pearson’s R; *p* = Significance level; c. Based on normal approximation; Nk = No, but I would like to know.

## Data Availability

The datasets generated and/or analyzed during the current study are not publicly available due to privacy and ethical restrictions but are available from the corresponding author on reasonable request.

## Abbreviations

The following abbreviations are used in this manuscript:

Q	Questions
N	Count
A	Age
G	Gender
B	Background
*p*	Significance
χ^2^	Pearson Chi-Square
WHO	World Health Organization
CDC	Centers for Disease Control and Prevention
IQ	Intelligence Coefficient
ADA	American Dental Association
NIDCR	National Institute of Dental and Craniofacial Research
FAN	Fluoride Action Network
